# Investigation of Repeat Client Drop-Out and Re-Enrolment Cycles in Fourteen Methadone Maintenance Treatment Clinics in Guangdong, China

**DOI:** 10.1371/journal.pone.0139942

**Published:** 2015-10-20

**Authors:** Lei Zhang, Xia Zou, Di Zhang, Xiaoling Li, Peizhen Zhao, Li Ling

**Affiliations:** 1 Sun Yat-sen Centre for Migrant Health Policy, Sun Yat-sen University, #74, Zhongshan Road II, Guangzhou, 510080, P.R. China; 2 The Kirby Institute, University of New South Wales, Sydney, NSW, Australia; 3 Faculty of Medical Statistics and Epidemiology, School of Public Health, Sun Yat-sen University, #74, Zhongshan Road II, Guangzhou, 510080, P.R. China; 4 Office of Medical Science, Sun Yat-sen University, Guangzhou, P. R. China; Medical University of Vienna, AUSTRIA

## Abstract

**Objective:**

Client adherence is vital for effective methadone maintenance treatment (MMT). This study explores the pattern and associated factors of client adherence, drop-out and re-enrolment in the Chinese MMT programme over the period of 2006–2013.

**Methods:**

This retrospective study was conducted in 14 MMT clinics in Guangdong Province, China. We employed Kaplan-Meier survival analysis to estimate the rates of drop-out and re-enrolment of MMT clients and multivariate Cox regression to identify associated factors.

**Results:**

Among 1,512 study participants, 79% have experienced ‘drop-out’ during the 7-year study period. However, 82% ‘dropped-out’ clients resumed treatment at a later time. Low education level (junior high or below versus otherwise, HR = 1.21, 1.05–1.40), low methadone dosage in the first treatment episode (<50 ml versus ≥50 ml, HR = 1.84, 1.64–2.06) and higher proportion of positive urine test (≥50% versus<50%, HR = 3.72, 3.30–4.20) during the first treatment episode were strong predictors of subsequent drop-outs of the participants. Among the ‘dropped-out’ clients, being female (HR = 1.40, 1.23–1.60), being married (HR = 1.19, 1.09–1.30), and having a higher proportion of positive urine tests in the first treatment episode (≥50% versus<50%, HR = 1.35, 1.20–1.51) had greater likelihood of subsequent re-enrolment in MMT. Clients receiving lower methadone dosage (first treatment episode <50 ml versus ≥50 ml, HR = 1.12, 1.03–1.23; the last intake before drop-out <50 ml versus ≥50 ml, HR = 1.16, 1.04–1.30) were also more likely to re-enrol.

**Conclusion:**

Persistent cycling in-and-out of clients in MMT programmes is common. Insufficient dosage and higher proportion of positive urine samples in the first treatment episode are the key determinants for subsequent client drop-out and re-enrolment. Interventions should target clients in their early stage of treatment to improve retention in the long term.

## Introduction

Methadone maintenance treatment (MMT) is a widely used opioid substitution treatment for drug users. By using methadone as a synthetic agent to block brain receptor affected by heroine and other opiates, the therapy is proven to effectively reduce the craving for drugs and clinical symptoms associated with withdrawal from opiates [1–3]. Numerous evidences demonstrated that MMT substantially reduce drug addiction, drug-related harms, crimes, risk behaviours and transmission of HIV [4–6]. Drug users received the treatment are marked by enhanced social productivities [7, 8], improved personal relationships [9, 10] and health status [11, 12]. However, MMT is not a curative therapy and requires sustaining administration of methadone in its clients. Treatment adherence often has direct influences on the treatment outcomes and is a main indicator for effectiveness of the programme [13].

MMT has been implemented in 63 countries around the world [14]. In China, the programme was launched as a major component of harm reduction programmes for drug users in 2003 [15–17]. In the subsequent year, China initiated its pilot MMT programme in eight clinics and later expanded to 761 clinics serving 407,000 drug users by the end of 2013 [18]. This covered approximately 16% of registered drug users in China [19]. The Chinese MMT programme has become one of the largest single drug treatment and care systems in the world. However, treatment adherence of its clients has been reportedly low. A recent systematic review indicates that over one-third of MMT clients terminate treatment within three month of their enrolment and only half of the clients retained in the programme in twelve months in China [20]. As MMT services in China are closely monitored by the public security, police raids and arrests for drug users near MMT sites are common [21–23], administrative detention and other structural factors have significantly interfered treatment and obscure the effects of MMT on durable behaviour change [24, 25]. Despite high drop-out rates, a large proportion of the dropped-out individuals were reportedly re- enrolled into the programme after a period of absence. Very little is known about the characteristics of the re-enrolled participants. Nevertheless, re-enrollment represents a valuable opportunity to ensure renewing reduction of harm and risk of infection of blood-borne diseases in the targeted population. We hypothesis that the re-enrolled patients may represent a specific subgroup of MMT participants who may differ from the drop-out individuals in terms of both demograhic and behavioural characteristics. The cycle of repeated dropping-out and returning likely forms a recognisable pattern over the course of treatment. Only a few studies investigated this phenomenon in China [26, 27].

Based on an ongoing follow-up study in fourteen MMT clinics in the Chinese province of Guangdong over the period of 2005–2013, this study investigates the pattern of client adherence, drop-out and re-enrolment in the Chinese MMT programme and associated factors. Understanding the cycling pattern in treatment may foster future implementation of the programme and tailor targeted interventions in those most needed to minimise drop-outs.

## Method

### Study sites

**Fig 1:**
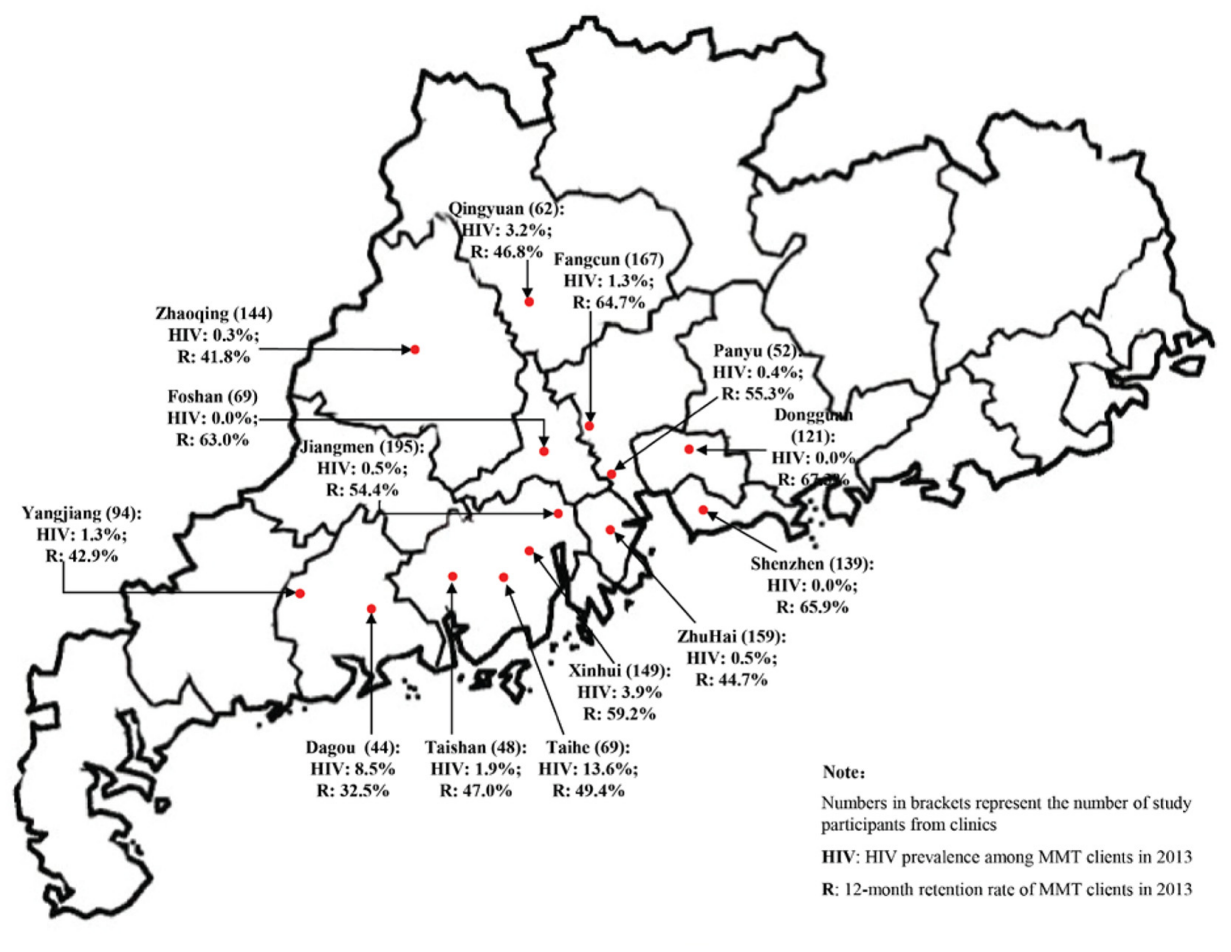
Geographical location, HIV prevalence and 12-month retention rates of study participants in 14 MMT clinics in Guangdong province in 2013.

Guangdong is one of the most economically developed provinces in Southern China and has more than 370,000 drug users, accounting for about one-sixth of all registered drug use in China [28]. The latest statistics indicated that 6.3%, 78.7% and 4.4% of drug users are infected with HIV, HCV and Tuberculosis (TB) in 2013, respectively [29]. A total of 61 MMT clinics have been established since the programme initiation in 2006. MMT clinics for this retrospective study were selected using stratified random sampling. All clinics in Guangdong province were firstly categorised into two groups based on whether the 1-year retention rate (defined as the proportion of participants retained within the first 12 months of enrolment) is above or below 50%. We randomly selected seven clinics from each category. A total of 14 clinics were established as our study sites (Fig 1).

### MMT provision

China has developed a national integrated web-based data system for MMT to collect client information. The database is administered by the National Centre for AIDS/STD Control and Prevention under the supervision of the Chinese Centre for Disease Control and Prevention. All participants received standardised MMT treatment including daily administration of methadone and regular urine testing.

Participants were required to pay ten RMB ($1.57 dollars) before receiving methadone in liquid solution under the supervision of clinic staff. The patients were subjected to urine testing on a random day per fortnight. Urine samples were tested for opioids (except methadone) using the immune-gold labelling technique (Shanghai Rongsheng Biotech Co., Ltd.). Blood samples were collected at baseline and in every 6 months to test for HIV, HCV, and syphilis infections. The staff in clinics monitored the daily dosage of methadone for the participants, the status of urine tests and infections. Referral to other MMT clinics can be obtained from the hosting clinics if participants were to transfer or relocate. Participants were referred to hospitals if they were diagnosed with TB infection or other clinical problems at enrolment.

### Recruitment of study participants

Participants who met the eligibility criteria for MMT and received the treatment in the selected clinics were recruited over the period of December 1st, 2011 and January 31th, 2012. The recruited participants must 1) have diagnosed opioid dependence according to the International Classification of Diseases-10 (ICD-10); 2) be aged 20 years or above; 3) be a local resident who settled in the catchment areas of the clinics; 4) have provided written informed consent for the use of their clinical data in MMT databases. We excluded clients only visit the clinics casually and those with incomplete treatment records.

### Data collection

Treatment records of recruited clients were collected both retrospectively (up to January 5th 2006) and prospectively (till March 12th 2014). We collected the following information from participants: (1) demographic information including age, gender, occupation, marital status, and education level; (2) baseline HIV and HCV status; (3) risk behaviours at the first enrolment; and (4) daily uptake dosage of methadone and (5) biweekly urine test results. Collected data was double-entered into the national MMT database and de-identified before analysis. We also collected the information related to their accessibility to MMT clinics, including travelling time and out-of-pocket payment.

### Measurements of study outcomes and definitions of prescribing characteristics

Primary indicators for treatment adherence were drop-out and re-enrolment rates of MMT clients. An individual was considered as ‘drop-out’ if he/she missed methadone intakes for 14 consecutive days or more. Participants were considered as “ever drop-outs” if only he/she drop out in their initial treatments or in the subsequent period of time during the study duration. Participants who did not drop-out were considered ‘retained’. Dropped- out individuals may be ‘loss-to-follow-up’ (LTFU) or return to treatment. LTFU clients were those left the programme for more than 14 days after the initial enrolment and did not return by the end of the study. For those who returned, they were classified as ‘re-enrolled’.

We defined the duration of treatment episode as the time from the enrolment (or re-enrolment) in MMT to the first day of following drop-out. We also explored the treatment gap days which defined as the durations from drop-outs to re-enrolments. The average methadone dosage was calculated as the total dosages divided by the duration of the treatment episodes. The positive urine testing rate was defined as the proportion of submitted urine samples that tested positive.

### Ethics approval

This study was approved by Ethics Review Committee at Sun Yat-sen University (Proposal number 71173245). All participants gave their written consent for their information to be stored in the national integrated web-based system for the MMT programme and the use of this study.

### Statistical analysis

We calculated the ever drop-out rate as the proportion of drop-outs among participants who have ever experience drop-out from MMT during the study duration. We calculate the times of drop-outs after the initial enrollment and each enrollment. Baseline information was compared across the retained, withdrawn, and re-enrolled groups using chi- square test for dichotomous variables. Kruskal Wallis H test was used to compare characteristics between retained, LTFU and re-enrolled groups. One-way ANOVA was used for the comparisons of normally distributed continuous variables. We employed Kaplan-Meier method to estimate the duration of the first treatment and calculate the gap days between the first drop-out and the subsequent re-enrolment, and the drop-out rates after one, two, and seven years of initial treatment. We explored independent correlates to the occurrence of drop-outs and re-enrolments using univariate Cox regression. Factors with *p* value less than 0.25 in univariate Cox regressions were included in multivariate analyses. We used multivariate Cox proportional hazard model to identify the predictors of drop-out [30], whereas Anderson and Gill model of Cox regression for counting process was employed to explore the predictors of re-enrolments [31]. Hazard ratios (*HRs*) were calculated with 95% confidence intervals (*CIs*). All analysis was conducted using SAS9.3 (SASInstitute Inc., Cary, NC, USA).

## Results

### Characteristics of study participants

A total of 1,512 MMT clients were included in this study. Of which, 1,194 (79.0%) had ever dropped out with 225 (18.8%) participants lost-to-follow-up and 969 (81.2%) re-enrolled later in the study period.

Participants had an average age 38.6 years (range 21–63), and was predominantly male (90.0%) and unemployed at first enrolment (63.2%). Just over half of the participants (53.4%) were divorced, widowed or single. Education level was low (junior high school or below: 81.2%, Table 1).

**Table 1:** Demographic characteristics, risk behaviour and treatment performance of 1,512 study participants, stratified in subgroups of retained, loss-to-follow-up, and re-enrolled clients (SD: standard deviation; IQR: interquartile range).

Characteristic	Total	Retained	LTFU	Re-enrolled	*Chi-2* test
	N = 1,512 (%)	N = 318 (%)	N = 225 (%)	N = 969 (%)	*P*-value
Age					
< 30	105 (6.9)	19 (6)	20 (8.9)	66 (6.8)	0.43
30~50	1360 (89.9)	286 (89.9)	198 (88.0)	876 (90.4)	
> 50	47 (3.1)	13 (4.1)	7 (3.1)	27 (2.8)	
mean(SD)	38.6 (5.99)	39.2 (6.07)	38.2 (6.32)	38.4 (5.88)	
Gender					
male	1361 (90.0)	280 (88.1)	208 (92.4)	873 (90.1)	0.24
female	151 (10.0)	38 (11.9)	17 (7.6)	96 (9.9)	
Occupation					
unemployed	956 (63.2)	206 (64.8)	149 (66.2)	601 (62.0)	0.41
employed	556 (36.8)	112 (35.2)	76 (33.8)	368 (38.0)	
Marital					
divorced/widowed/single	807 (53.4)	157 (49.4)	121 (53.8)	529 (54.6)	0.27
married	705 (46.6)	161 (50.6)	104 (46.2)	440 (45.4)	
Education					
junior high and below	1228 (81.2)	252 (79.2)	177 (78.7)	799 (82.5)	0.41
senior high and above	284 (18.8)	66 (20.8)	48 (21.3)	170 (17.5)	
HIV Status					
positive	141 (9.3)	28 (8.8)	23 (10.2)	90 (9.3)	0.86
negative	1361 (90.0)	290 (91.2)	202 (89.8)	869 (89.7)	
missing	10 (0.7)	0 (0.0)	0 (0.0)	10 (1.0)	
HCV Status					
positive	1254 (82.9)	273 (85.8)	182 (80.9)	799 (82.5)	0.33
negative	242 (16.0)	43 (13.5)	40 (17.8)	159 (16.4)	
missing	16 (1.1)	2 (0.6)	3 (1.3)	11 (1.1)	
Duration of addiction					
< 5	164 (10.8)	33 (10.4)	19 (8.4)	112 (11.6)	0.11
5~15	869 (57.5)	178 (56.0)	125 (55.6)	566 (58.4)	
> 15	479 (31.7)	107 (33.6)	81 (36.0)	291 (30.0)	
mean(SD)	12.2 (5.13)	12.5 (5.13)	12.7 (5.25)	12.0 (5.09)	
Types of drug abuse					
heroin	1482 (98.0)	314 (98.7)	220 (97.8)	948 (97.8)	0.58
other	30 (2.0)	4 (1.3)	5 (2.2)	21 (2.2)	
Inject drugs in the past 30 days					
yes	1039 (68.7)	231 (72.6)	158 (70.2)	650 (67.1)	0.16
no	228 (15.1)	56 (17.6)	44 (19.6)	128 (13.2)	
missing	245 (16.2)	31 (9.7)	23 (10.2)	191 (19.7)	
Share needle-syringe in past 30 days					
yes	89 (8.6)	14 (6.1)	16 (10.1)	59 (9.1)	0.26
no	945 (90.9)	217 (93.9)	139 (88.0)	589 (90.6)	
missing	5 (0.5)	0 (0.0)	3 (1.9)	2 (0.3)	
Travelling time to clinics (minutes)					
0~	202 (13.4)	47 (14.8)	27 (12)	128 (13.2)	0.62
15~	1309 (86.5)	270 (85.2)	198 (88.0)	841 (86.8)	
missing	1 (0.1)	1 (0.3)	0 (0.0)	0 (0.0)	
Average methadone dosage in the first treatment duration (ml)					
≥50	789 (52.2)	214 (67.3)	115 (51.1)	460 (47.5)	<0.01
<50	723 (47.8)	104 (32.7)	110 (48.9)	509 (52.5)	
mean (SD)	55.5 (26.5)	64.2 (28.0)	55.4 (25.7)	52.7 (25.5)	
The last methadone dosage (ml)					
≥50	315 (20.8)	67 (21.7)	35 (15.6)	213 (22.0)	<0.01
<50	1197 (79.2)	251 (78.9)	190 (84.4)	756 (78.0)	
mean (SD)	36.6 (27.9)	37.5 (28.3)	31.5 (21.9)	37.5 (28.9)	
Positive rate for urine tests (%)					
≥50	557 (36.8)	37 (11.6)	68 (30.2)	452 (46.7)	<0.01
<50	862 (57.1)	269 (84.6)	143 (63.6)	450 (46.5)	
missing	93 (6.2)	12 (3.8)	14 (6.2)	67 (6.9)	

### Treatment performance and adherence

The average methadone dosage in the first treatment episode was 55.5 (standard deviation, SD = 26.5) millilitres among all participants, among whom, the dosage in the retained group was the highest (retained: 64.2±28.0, LTFU: 55.4±25.7, re-enrolled: 52.7±25.5, Kruskal Wallis, *p<*0.01). Among clients who experienced drop-out, LTFU clients had significantly lower dosage than returned clients in the last administration before drop-out (LTFU: 31.5±21.9, re-enrolled: 37.5±28.9, Kruskal Wallis, *p<*0.01).

Overall, 557 (36.8%) participants were urine tested positive in more than 50% of testing occasions. This rate was lowest in the retained group (11.6%), followed by the LTFU group (30.2%) but highest among the re-enrolled group (46.7%) (Chi-2 test, *p<*0.01, Table 1).

**Fig 2:**
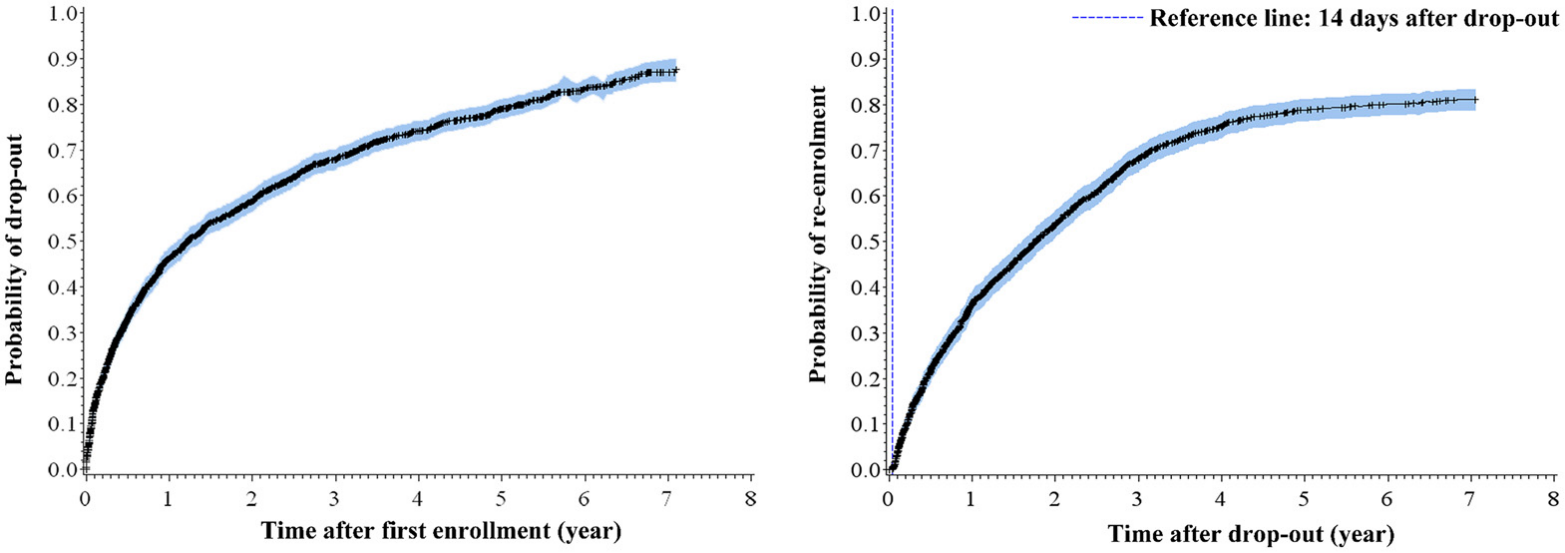
Kaplan-Meier failure curve for (a) probability of drop-out after first enrolment; (b) probability of re-enrolment from the onset of first drop-out.

The cumulative probability of drop-out events increased with time. Estimated 46.3%, 58.8% and 87.6% study participants experienced drop-out after one, two and seven year of initial enrolment (Fig 2a). Cumulative probability of re-enrolment also demonstrated similar trend. Estimated 36.1%, 53.8% and 81.2% dropped-out individuals returned to treatment in one, two and seven years (Fig 2b).

**Fig 3:**
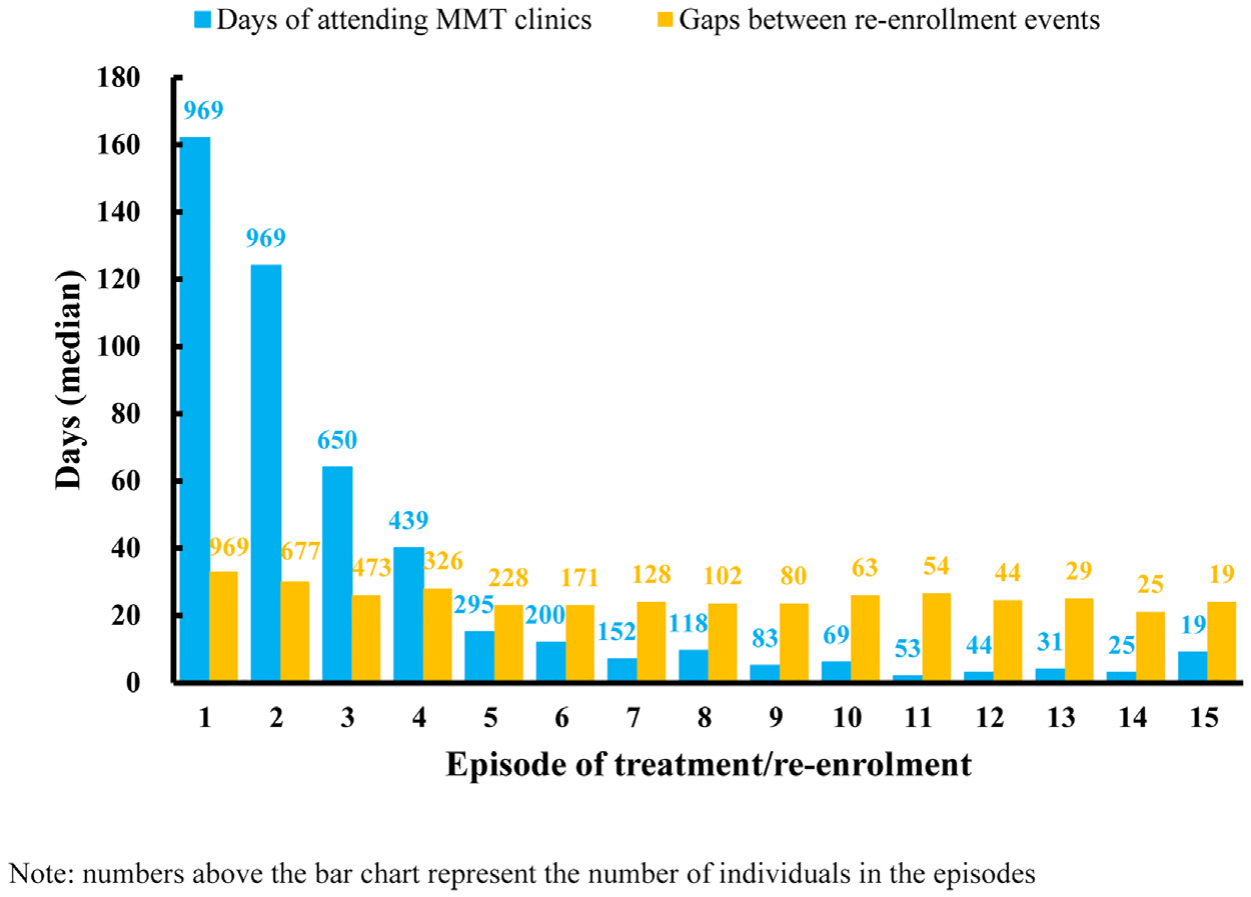
Duration of MMT participation and gaps between dropout and re-enrolment events.

The median duration of the first treatment episode was 162 (interquartile range: 35–499) days among re- enrolled individuals. The duration of the following episodes then declined substantially (2nd: 124 [17–536]; 3rd: 64 [7–35]; 4th: 40 [4–239]). Clients could not sustain treatment more than 30 days beyond the 4th episode. In comparison, the duration of treatment interruption (onset 14 days after the last recorded day of attendance) consistently varies between 21 to 33 days regardless of treatment episode (Fig 3).

### Predictors of drop-out and re-enrolment

**Fig 4:**
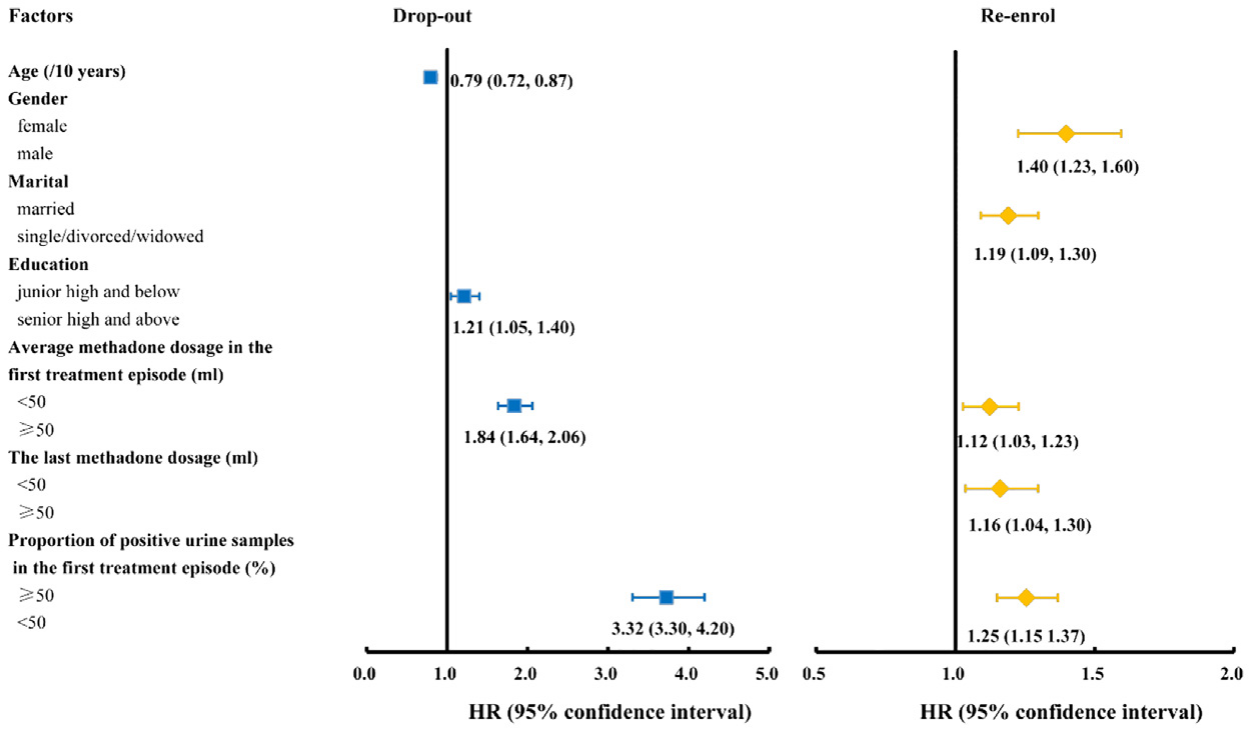
Associated factors of drop-out and re-enrolments of MMT participants based on multivariate Cox regression.

Multivariate Cox regression analysis indicated that the likelihood of drop-out reduced significantly by 20.6% (12.8–27.7%) with every 10-year increase in age. Low education level (junior high or below versus otherwise, *HR*: 1.21, 1.05–1.40), low methadone dosage during the first treatment episode (<50 ml versus ≥50 ml, *HR*: 1.84, 1.64–2.06) and higher proportion of positive urine test (≥50% versus<50%, *HR*: 3.72, 3.30–4.20) in first treatment episode were strong predictors of subsequent drop-outs of the participants (S1 Table, Fig 4).

Among participants who experienced dropped out, being female (*HR*: 1.40, 1.23–1.60), being married (*HR*: 1.19, 1.09–1.30), and having higher proportion of positive urine samples in the first treatment episode (≥50% versus<50%, *HR*: 1.35, 1.20–1.51) had greater likelihood of re-enrolment in MMT. Clients received lower methadone dosage (first treatment episode <50 ml versus ≥50 ml, *HR*: 1.12, 1.03–1.23; the last intake prior to last drop-out <50 ml versus ≥50 ml, *HR*: 1.16, 1.04–1.30) were more likely to re-enrol (S1 Table, Fig 4).

## Discussion

This study highlights a typical pattern of repeated drop-out and re-entry to treatment in the majority of MMT clients in China. This phenomenon of continuous cycling-in-and-out of MMT clients has been previously reported in developed country settings [32, 33], but is first in China, where MMT has just been established over a decade [34]. We reported a median first treatment duration of 6-month among re- enrolled clients and retaining clients in the following treatment episodes became increasingly difficult.

Investigation of underlying factors for drop-out and re-entry has revealed significant associations with client demographic characteristics and treatment performance during the first treatment episode.

MMT adherence has been extensively studied in China. A recent systematic review has indicated that relapsing drug-use, criminal activities, police detention and self-withdrawal in sum account for the majority of MMT drop-outs [20]. Whereas most previous studies fall short in distinguishing individuals who experienced temporarily service disruption and those determine to leave the programme, this study has indicated that most ‘drop-outs’ are short-term with clients repeatedly re-entering the programme multiple times. Relapsing drug-use is common in early phase of treatment [35] and in the Chinese setting this often leads to subsequent police detention and mandatory detoxification [36]. Consistently, our study has shown that individuals with more than 50% positive urine samples during the first treatment episode are substantially associated with treatment drop-out. The causes of relapsing can be multi-fold, but numerous evidences have indicated that insufficient methadone dosage is a key underlying reason [37]. This is aligned with the finding that average methadone dosage below 50 ml/day in the first treatment episode has increased the likelihood of client drop-outs by more than 50% in our study. Misconception about MMT remained common, even among health professionals. Many regard MMT as a curative therapy and since methadone is an opioid substitution its provision should be kept to minimal [38].

We found that approximately one-fifth of clients were lost-to-follow-up during the course of treatment. In comparison with clients who voluntarily discharge from MMT in developed country settings [39], these withdrawn clients in the Chinese settings often leave without notifying programme administration. They cannot be traced or followed-up in anyway. Some common reasons for the loss-to-follow-up include death, transfer to other clinics and relocation. Misconception about MMT among Chinese drug users is also well- documented. Individuals may enter MMT with the expectation of a quick fix of their addictive issues, but after learning about MMT a sustaining and likely long-term treatment procedure, they are often disappointed [40, 41]. In addition, with clearly alleviation in addictive syndromes and improvements in physical strengthen (consistently lower last MMT dosage among LTFU clients in our study), MMT clients may consider themselves fit enough to leave the programme [40, 42]. This is consistent with a higher drop- out rate among poorly educated clients in our study. Poor education often contributes to the misconception about MMT and self-perceived successful detoxification, leading to immature withdrawal from the programme [40, 41, 43].

Despite high drop-out rate (79%) among MMT participants, most clients (~80%) choose to return to MMT after a short period of ‘drop-out’. The underlying reason for this ongoing drop-out and re-enrollment pattern could be multi-fold. MMT participants in China had common misconceptions towards the treatment programme. They regard MMT as a transient programme for drug detoxification. Most participants did not want to retain on treatment once their addictive reactions are alleviated [41]. However, once they leave MMT but only experience the abstinence symptoms again and cannot afford opioids, they return to MMT. In addition, MMT participants who used drugs during the treatment course may choose to stay away from the programme temporarily in the fear of a positive urine test, which could potentially result in police arrest. They appear not to be able to keep a regular and sustaining attendance to the clinic, but certainly did not intend to terminate the service completely either. Among ‘dropped-out’ clients, females and married individuals are more likely to return to treatment. Females are known to be more self-aware of their own health status [44] and married individuals may receive better family and financial support [45]. The cycling pattern of MMT clients is marked by a declining duration of treatment episodes but relatively constant interruption of 3–4 weeks.

Consistent with the regression trend for drop-outs, clients received substantially higher dosage and being urine tested positive more frequently during the first treatment episode predict a higher re-entry probability among drop-outs. That is, performance during the first treatment episode has decisive effects on the latter adherence of clients. It also becomes harder to sustain effective treatment if individual clients have developed the habit of cycling in-and-out the treatment programme. The finding also suggests a greater chance of treatment resume among relapsed drug users or those continuing to use drugs while on treatment. Their treatment is likely deterred by ongoing drug use/relapse due to insufficient methadone dosage. However, they choose to return to treatment often when they can no longer afford drugs and with MMT their addictive reactions can at least be temporarily released [24].

A number of study limitations should be noted. First, we adapt the definition of drop-out as 14-day absence based on guidelines from China CDC and past literature. However, there has been ongoing debate about whether a 30-day threshold should be used [41]. Second, we could not conduct follow-up studies among those who have lost to follow-up. There has been very little information about this subgroup apart from their limited demographic information at enrolment and treatment status. Third, this study has a long enrolment period of seven years and diversified study sites, demographic characteristics, risk behaviours and treatment performance may vary both temporally and geographically.

We investigated a very important behavioural pattern of MMT clients cycling in-and-out the service in China. Conclusively, drop-out rate after the first treatment episode may not reflect the actual adherence of MMT clients, as the majority of clients retain or return to treatment after a brief gap of 3–4 weeks. This calls for effective interventions to reduce the frequency of repeated enrolment of the programme.

Improvement on methadone dosage with a more customised adjustment scheme, monitoring of additive reactions and health education to reduce misconception on MMT are necessary for the provision of sustaining treatment to drug users. Further exploratory follow-up study of LTFU clients is required to understand the underlying causes of treatment termination. Staff plays a critical role in retaining the participants in the treatment. Further studies on the impacts of the MMT staff on the drop-out are necessary to explore this.

## Conclusion

Persistent cycling in-and-out of clients in MMT programme is common. Insufficient dosage and higher proportion of positive urine samples in the first treatment episode are the key determinant for subsequent client drop-out and re-enrolment. Interventions should target clients in their early stage of treatment to improve retention in the long term.

## Supporting Information

S1 Table Results of univariate and multivariate Cox regression analysis for individuals who retained or experienced drop-out (loss-to-follow-up and re-enrolled) in 14 MMT clinics in China. (DOCX)
