# Supplementary material for: Investigation of Repeat Client Drop-Out and Re-Enrolment Cycles in Fourteen Methadone Maintenance Treatment Clinics in Guangdong, China
Source: PLoS One. 2015 Oct 20;10(10):e0139942. doi: 10.1371/journal.pone.0139942 (PMC4618733; doi:10.1371/journal.pone.0139942)
Supplement: S1 Table — (DOCX) [file pone.0139942.s001.docx]

**S1 Table. Results of univariate and multivariate Cox regression analysis for individuals who retained or experienced drop-out (loss-to-follow-up and re-enrolled) in 14 MMT clinics in China**

| **Factors** | **Dropped-out (re-enrollment / withdraw) vs. Retained§** | | | | | | | |  | **Re-enrolled vs. LTFU¶** | | | | | | | |
| --- | --- | --- | --- | --- | --- | --- | --- | --- | --- | --- | --- | --- | --- | --- | --- | --- | --- |
|  | **Univariate** | | |  | **Multivariate** | | | |  | **Univariate** | | |  | **Multivariate** | | | |
|  | ***HR*** | ***LCL*** | ***UCL*** |  | ***HR*** | ***LCL*** | ***UCL*** | ***P*** |  | ***HR*** | ***LCL*** | ***UCL*** |  | ***HR*** | ***LCL*** | ***UCL*** | ***P*** |
| **Age (/10 years)** | 0.897 | 0.817 | 0.985† |  | 0.794 | 0.723 | 0.872 | <0.0001 |  | 1.005 | 0.949 | 1.065 |  |  |  |  |  |
| **Gender** |  |  |  |  |  |  |  |  |  |  |  |  |  |  |  |  |  |
| female | 0.899 | 0.735 | 1.099 |  |  |  |  |  |  | 1.353 | 1.212 | 1.510* |  | 1.398 | 1.225 | 1.595 | <0.0001 |
| male |  |  |  |  |  |  |  |  |  |  |  |  |  |  |  |  |  |
| **Marital** |  |  |  |  |  |  |  |  |  |  |  |  |  |  |  |  |  |
| married | 0.864 | 0.771 | 0.968† |  |  |  |  |  |  | 1.13 | 1.054 | 1.212* |  | 1.190 | 1.091 | 1.298 | <0.0001 |
| single/divorced/widowed |  |  |  |  |  |  |  |  |  |  |  |  |  |  |  |  |  |
| **Employment** |  |  |  |  |  |  |  |  |  |  |  |  |  |  |  |  |  |
| employed | 1.040 | 0.925 | 1.169 |  |  |  |  |  |  | 1.114 | 1.037 | 1.196* |  |  |  |  |  |
| unempolyed |  |  |  |  |  |  |  |  |  |  |  |  |  |  |  |  |  |
| **Education** |  |  |  |  |  |  |  |  |  |  |  |  |  |  |  |  |  |
| junior high and below | 1.179 | 1.018 | 1.366† |  | 1.211 | 1.045 | 1.403 | 0.0109 |  | 1.083 | 0.989 | 1.186‡ |  |  |  |  |  |
| senior high and above |  |  |  |  |  |  |  |  |  |  |  |  |  |  |  |  |  |
| **HIV** |  |  |  |  |  |  |  |  |  |  |  |  |  |  |  |  |  |
| positive | 1.097 | 0.904 | 1.332 |  |  |  |  |  |  | 0.979 | 0.867 | 1.105 |  |  |  |  |  |
| negative |  |  |  |  |  |  |  |  |  |  |  |  |  |  |  |  |  |
| **HCV** |  |  |  |  |  |  |  |  |  |  |  |  |  |  |  |  |  |
| positive | 0.916 | 0.787 | 1.067 |  |  |  |  |  |  | 1.022 | 0.93 | 1.122 |  |  |  |  |  |
| negative |  |  |  |  |  |  |  |  |  |  |  |  |  |  |  |  |  |
| **The years of drug use** |  |  |  |  |  |  |  |  |  |  |  |  |  |  |  |  |  |
| < 5 | 1.076 | 0.882 | 1.313 |  |  |  |  |  |  | 1.014 | 0.898 | 1.144 |  |  |  |  |  |
| 5~15 | 1.001 | 0.882 | 1.135 |  |  |  |  |  |  | 0.955 | 0.883 | 1.033‡ |  |  |  |  |  |
| ≥ 15 |  |  |  |  |  |  |  |  |  |  |  |  |  |  |  |  |  |
| **Types of drug abuse** |  |  |  |  |  |  |  |  |  |  |  |  |  |  |  |  |  |
| heroin | 1.066 | 0.554 | 2.054 |  |  |  |  |  |  | 1.528 | 0.962 | 2.428‡ |  |  |  |  |  |
| other |  |  |  |  |  |  |  |  |  |  |  |  |  |  |  |  |  |
| **Inject drugs in the past 30 days** | | |  |  |  |  |  |  |  |  |  |  |  |  |  |  |  |
| yes | 1.045 | 0.886 | 1.232 |  |  |  |  |  |  | 0.896 | 0.809 | 0.992† |  |  |  |  |  |
| no |  |  |  |  |  |  |  |  |  |  |  |  |  |  |  |  |  |
| **Share needle-syringe in past 30 days** | | |  |  |  |  |  |  |  |  |  |  |  |  |  |  |  |
| yes | 1.08 | 0.851 | 1.37 |  |  |  |  |  |  | 1.097 | 0.951 | 1.265‡ |  |  |  |  |  |
| no |  |  |  |  |  |  |  |  |  |  |  |  |  |  |  |  |  |
| **Travelling time to clinics (minutes)** | | |  |  |  |  |  |  |  |  |  |  |  |  |  |  |  |
| <15 | 0.987 | 0.833 | 1.168 |  |  |  |  |  |  | 0.993 | 0.84 | 1.037‡ |  |  |  |  |  |
| ≥15 |  |  |  |  |  |  |  |  |  |  |  |  |  |  |  |  |  |
| **Average methadone dosage in the first treatment episode (ml)** | | | | | |  |  |  |  |  |  |  |  |  |  |  |  |
| <50 | 1.656 | 1.479 | 1.855* |  | 1.835 | 1.635 | 2.058 | <0.0001 |  | 1.098 | 1.025 | 1.176* |  | 1.123 | 1.027 | 1.227 | 0.0105 |
| ≥50 |  |  |  |  |  |  |  |  |  |  |  |  |  |  |  |  |  |
| **The last methadone dosage (ml)** | | |  |  |  |  |  |  |  |  |  |  |  |  |  |  |  |
| <50 | 0.917 | 0.797 | 1.054‡ |  |  |  |  |  |  | 1.137 | 1.048 | 1.233* |  | 1.160 | 1.036 | 1.297 | 0.0098 |
| ≥50 |  |  |  |  |  |  |  |  |  |  |  |  |  |  |  |  |  |
| **Proportion of positive urine samples in the first treatment episode (%)** | | | | | | | |  |  |  |  |  |  |  |  |  |  |
| ≥50 | 3.47 | 3.082 | 3.906* |  | 3.724 | 3.304 | 4.198 | <0.0001 |  | 1.224 | 1.142 | 1.312* |  | 1.254 | 1.15 | 1.368 | <0.0001 |
| <50 |  |  |  |  |  |  |  |  |  |  |  |  |  |  |  |  |  |

Univariate Cox regression: *P<0.01, †P<0.05, ‡P<0.25

Methods for survival analysis: § Cox proportional hazard regression model; ¶ Anderson and Gill (AG) model for recurrent event data.
